# Palladium–platinum core-shell icosahedra with substantially enhanced activity and durability towards oxygen reduction

**DOI:** 10.1038/ncomms8594

**Published:** 2015-07-02

**Authors:** Xue Wang, Sang-Il Choi, Luke T. Roling, Ming Luo, Cheng Ma, Lei Zhang, Miaofang Chi, Jingyue Liu, Zhaoxiong Xie, Jeffrey A. Herron, Manos Mavrikakis, Younan Xia

**Affiliations:** 1 Wallace H. Coulter Department of Biomedical Engineering, Georgia Institute of Technology and Emory University, Atlanta, Georgia 30332, USA; 2 State Key Laboratory of Physical Chemistry of Solid Surfaces, Collaborative Innovation Center of Chemistry for Energy Materials, Xiamen University, Xiamen, Fujian 361005, China; 3 Department of Chemistry, Xiamen University, Xiamen, Fujian 361005, China; 4 Department of Chemical and Biological Engineering, University of Wisconsin-Madison, Madison, Wisconsin 53706, USA; 5 Center for Nanophase Materials Sciences, Oak Ridge National Laboratory, Oak Ridge, Tennessee 37831, USA; 6 Department of Physics, Arizona State University, Tempe, Arizona 85287, USA; 7 School of Chemistry and Biochemistry, Georgia Institute of Technology, Atlanta, Georgia 30332, USA; 8 School of Chemical and Biomolecular Engineering, Georgia Institute of Technology, Atlanta, Georgia 30332, USA

## Abstract

Conformal deposition of platinum as ultrathin shells on facet-controlled palladium nanocrystals offers a great opportunity to enhance the catalytic performance while reducing its loading. Here we report such a system based on palladium icosahedra. Owing to lateral confinement imposed by twin boundaries and thus vertical relaxation only, the platinum overlayers evolve into a corrugated structure under compressive strain. For the core-shell nanocrystals with an average of 2.7 platinum overlayers, their specific and platinum mass activities towards oxygen reduction are enhanced by eight- and sevenfold, respectively, relative to a commercial catalyst. Density functional theory calculations indicate that the enhancement can be attributed to the weakened binding of hydroxyl to the compressed platinum surface supported on palladium. After 10,000 testing cycles, the mass activity of the core-shell nanocrystals is still four times higher than the commercial catalyst. These results demonstrate an effective approach to the development of electrocatalysts with greatly enhanced activity and durability.

## Abstract (web summary)

Core-shell catalysts can enhance activity while reducing the loading of expensive catalyst materials. Here, the authors report a palladium@platinum system in which the platinum shells evolve into a corrugated structure with compressive strains, with subsequent enhancement of oxygen reduction activity.

## Graphical abstract

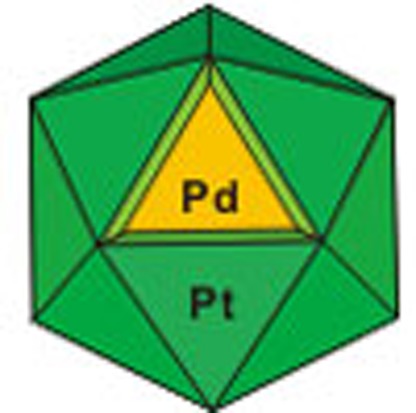

Platinum is a critical component of the catalysts for both the oxygen reduction reaction (ORR) and hydrogen oxidation reaction involved in a proton-exchange membrane fuel cell^1^. Owing to the sluggish kinetics of ORR, a large amount of Pt has to be deposited on the cathode in order to maintain the performance of such a device over a long period of time^2^. Because of its low abundance in the earth's crust and the ever-increasing demand from the automotive industry, the materials cost associated with a Pt-based catalyst has made it impractical to commercialize the proton-exchange membrane fuel cell technology at an industrial scale^3^. One solution to this problem is to increase the utilization efficiency of Pt by reducing the particle size and thus increasing the dispersion of Pt atoms^4^. For this reason, the commercial ORR catalyst is typically based on Pt particles of only a few nanometres in size. Despite the extensive use of these small Pt particles, it has been very difficult to optimize their specific activity by controlling the type of facet expressed on the surface. In addition, such tiny particles tend to aggregate and/or be detached from the carbon support, leading to major deterioration in performance over time during device operation^5^.

In recent years, a number of alternative strategies have been explored to increase the utilization efficiency of Pt by simultaneously increasing the dispersion of Pt atoms and enhancing the specific activity^6^,^7^,^8^,^9^,^10^,^11^,^12^,^13^,^14^,^15^,^16^,^17^,^18^,^19^,^20^,^21^,^22^,^23^. One approach is based on the conformal deposition of Pt atoms as ultrathin shells on the surfaces of nanoparticles made of a less expensive and/or a more abundant metal^7^,^8^,^9^,^10^,^11^,^12^,^13^,^14^. To this end, both chemical and electrochemical methods have been developed to generate Pt conformal shells of only a few atomic layers on Pd-based nanoparticles. Enhancement in mass activity towards ORR has been demonstrated for the resultant bimetallic nanoparticles. However, the Pd nanoparticles involved in most of these studies were either polycrystalline in structure or enclosed by facets that do not favour ORR.

It is well-established that the specific activity of Pt(111) towards ORR is at least twice that of Pt(100) (ref 24). It has also been demonstrated that the introduction of surface strain can dramatically modify the binding properties of adsorbates and thus their chemical reactivity^25^. By introducing compressive strain into the Pt lattice to weaken the binding of adsorbed O and OH, the ORR specific activity of Pt(111) can be enhanced^26^; the optimal compression was reported to be between 2 and 3%^27^,^28^. In addition, Yang and coworkers recently reported that the specific activity of Pt_3_Ni icosahedra, whose surface was under a tensile strain, was 1.5 times that of Pt_3_Ni octahedra with a similar size, even though both of their surfaces were covered by {111} facets^23^. Taken together, we believed that the specific activity of an ORR catalyst could be further enhanced by switching to nanocrystals with both {111} facets and twin defects on their surfaces.

Here we validate this hypothesis using Pd@Pt_nL_ (where the number of Pt overlayers, *n*=0.7–4.3) icosahedra as a typical example, in which the thickness of the Pt shells can be controlled down to a single atomic layer. Owing to a tensile strain on the surface of a Pd icosahedral seed, extra Pt atoms are introduced into the overlayers during conformal deposition. Under the lateral confinement imposed by the boundaries of twin defects, the Pt overlayers are forced to evolve into a compressed, corrugated structure through relaxation normal to the surface. The Pd@Pt_nL_ icosahedra show remarkable improvements in terms of both activity and durability when benchmarked against a commercial Pt/C catalyst. Self-consistent density functional theory (DFT) calculations suggest that the enhancement in specific activity can be attributed to the weakened OH binding, as a result of the uniquely strained Pt overlayers and the ligand interactions with the underlying Pd substrate.

## Results

### Structural characterization of the Pd@Pt_nL_ icosahedra

The Pd icosahedral seeds had an average diameter (*d*) of 13.4±3.2 nm (Supplementary Fig. 1). They were prepared by slightly modifying a protocol developed by our group^29^. The Pd@Pt_nL_ icosahedra were synthesized using a protocol we recently demonstrated for the atomic layer-by-layer deposition of Pt on Pd nanocubes and octahedra^13^,^14^. Specifically, we used a syringe pump to introduce a Na_2_PtCl_6_ precursor solution in ethylene glycol into a growth solution (also, ethylene glycol based) containing the Pd icosahedral seeds, poly(vinyl pyrrolidone) (PVP), ascorbic acid and KBr. On addition into the growth solution, the precursor should be immediately reduced by ascorbic acid and ethylene glycol to generate Pt atoms. At an injection rate of 4 ml h^−1^, the newly formed Pt atoms could be maintained at a relatively low concentration in the growth solution to prevent them from self-nucleation and thereby ensure that all of them would be deposited on the Pd seeds. By conducting the synthesis at 200 °C, we could accelerate the surface diffusion of the Pt adatoms to generate a conformal, ultrathin shell on the surface of each Pd seed.

**Figure 1:**
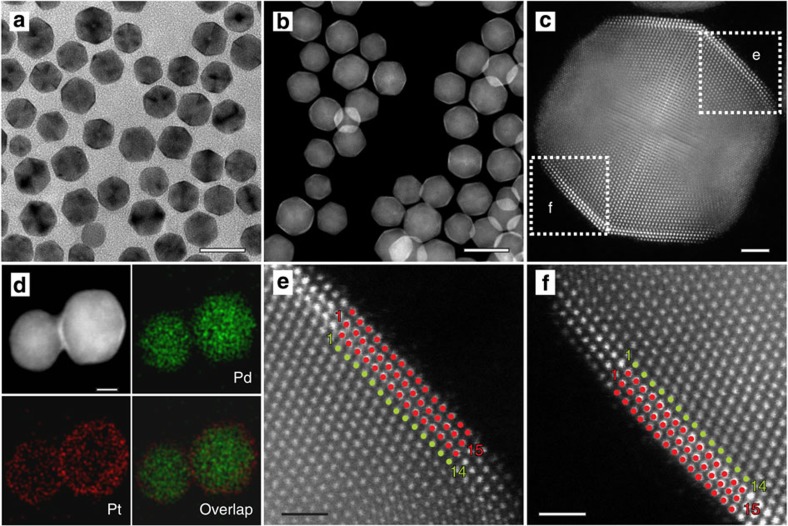
Structural and compositional analyses of the Pd@Pt_2.7L_ icosahedra. (**a**) TEM and (**b**) HAADF-STEM images. Scale bar, 20 nm. (**c**) Atomic-resolution HAADF-STEM image taken from a single particle along a two-fold symmetry axis, showing three atomic layers of Pt in the shell (with a brighter contrast) and the Pd atoms in the core. Scale bar, 2 nm. (**d**) HAADF-STEM image of two Pd@Pt_2.7L_ icosahedra and the corresponding energy-dispersive X-ray spectroscopy mapping of Pd and Pt, confirming a core-shell structure. Scale bar, 5 nm. (**e**,**f**) Atomic-resolution HAADF-STEM images taken from the edges marked by boxes in (**c**), revealing the detailed arrangements of Pd and Pt atoms (green dots: Pd atoms; red dots: Pt atoms). Scale bar, 1 nm.

Figure 1a–f, shows electron microscopy images of the Pd@Pt_nL_ core-shell icosahedra obtained after adding 16 ml of the Pt precursor solution (0.06 mg ml^−1^). Specifically, Fig. 1a,b, shows transmission electron microscopy (TEM) and high-angle annular dark-field scanning TEM (HAADF-STEM) images of the sample, indicating that the icosahedral shape was well preserved during the deposition of Pt atoms. The average diameter of the icosahedra increased to 14.9±2.3 nm, corresponding to a shell thickness of 0.75 nm. Figure 1c shows an aberration-corrected HAADF-STEM image taken from a single Pd@Pt_nL_ icosahedron along one of its two-fold symmetry axes^30^, confirming that the Pt atoms were indeed deposited as a conformal, uniform shell on the surface of a Pd icosahedral seed. The contrast between the Pt shell (brighter) and the Pd core can be attributed to the large difference in atomic number between these two elements. The well-resolved twin planes indicate that the core-shell nanocrystal still had a multiply twinned structure consistent with that of an icosahedron. The image suggests that the Pt shell was only three atomic layers in thickness. We also determined the average number of Pt atomic layers by inductively coupled plasma mass spectrometry (ICP-MS) and obtained a value of 2.7 (Table 1). Since the number of Pt atomic layers derived from the ICP-MS analysis represents the average value from a very large number of particles, it is used exclusively in our discussion unless otherwise specified. We denote the sample of core-shell nanocrystals shown in Fig. 1 as Pd@Pt_2.7L_ icosahedra. We further analysed the bimetallic nanocrystals by energy-dispersive X-ray spectroscopy (EDX) mapping to confirm the formation of a Pd–Pt core-shell structure (Fig. 1d).

From the atomic-resolution HAADF-STEM images shown in Fig. 1e,f, we found an interesting new phenomenon: the packing of Pt atoms in the overlayers did not exactly follow the underlying Pd atoms of the core. Instead, the Pt atoms underwent relaxation to take a corrugated structure (Supplementary Fig. 2). The formation of such a corrugated structure could be attributed to the existence of both tensile strain and twin boundaries on the surface of a Pd icosahedral seed. An icosahedron comprises 20 tetrahedral subunits, with 20 {111} facets and 30 twin boundaries on its surface. Each {111} facet is laterally confined by three twin boundaries. Molecular dynamics simulations have shown that the atomic lattice near the surface of an icosahedron is slightly stretched relative to that in the bulk^23^. This lattice expansion allows for extra Pt atoms to be packed into each Pt overlayer relative to the number of Pd atoms on the surface of the initial seed. The introduction of extra Pt atoms into the overlayers creates a compressive strain on the Pt shell, even though the strain in the underlying Pd layer is tensile. As a result of this compressive strain and lattice mismatch, surface relaxation must be involved during the deposition of a conformal Pt shell on a Pd icosahedral seed. Since lateral relaxation on the {111} facet is constrained by the twin boundaries, the Pt atoms can only relax along a direction normal to the surface, generating a corrugated structure.

By reducing the volume of the precursor solution added while fixing the amount of the Pd icosahedral seeds, the Pt shell thickness could be readily tuned from multiple layers down to a single atomic layer or even below. As indicated by the ICP-MS data in Table 1, the number of the Pt atomic layers was reduced to 2 and 0.7 (that is, Pd@Pt_2L_ and Pd@Pt_0.7L_, respectively, Supplementary Fig. 3) when the volume of the precursor solution was decreased from 16 to 12 and 4 ml, respectively. A corrugated surface was also observed for the sample of Pd@Pt_2L_ icosahedra (Supplementary Fig. 3c). In comparison, the core-shell icosahedra showed a flat surface when the Pt shell was less than one atomic layer in thickness (see Supplementary Fig. 3g for Pd@Pt_0.7L_). If the precursor solution was fixed in volume, the thickness of the Pt shell could be increased up to 4.3 atomic layers by reducing the number of Pd icosahedral seeds involved. Interestingly, the Pt deposition efficiency could be maintained at a level around 88% for all these syntheses (Supplementary Table 1).

The relative rates for atom deposition (*V*_depo_) and surface diffusion (*V*_diff_) are critical to the generation of a conformal, uniform Pt shell on the surface of a Pd icosahedral seed^31^. Owing to the use of a relatively strong reducing agent like ascorbic acid, the Pt(IV) precursor was supposed to be reduced immediately on its introduction into the growth solution. As a result, *V*_depo_ was largely determined by the injection rate of the precursor solution. For an icosahedral seed, every vertex is intersected by five twin boundaries. Therefore, the Pt atoms tend to be initially deposited on the vertices of a Pd icosahedral seed and the adatoms then diffuse to the edges and side faces. Owing to the use of a slow injection rate for the Pt precursor (that is, small *V*_depo_) and high reaction temperature (and thus large *V*_diff_), the Pt atoms deposited on the vertices could quickly diffuse to the edges and side faces to generate a conformal, uniform shell. The deposition of Pt atoms indeed followed a layer-by-layer growth mode, leading to the formation of Pd@Pt_nL_ icosahedra with a well-controlled thickness for the shell. When the reaction temperature was reduced to 120 °C, the surface diffusion of Pt adatoms was decelerated and most of the deposited Pt atoms tended to stay at the vertices and edges, resulting in the formation of icosahedra with concave surfaces (Supplementary Fig. 4a). When the reaction temperature was increased to 140 °C, surface diffusion would be accelerated for the deposited Pt atoms. As such, the extent of concaveness of the core-shell icosahedra would become less significant (Supplementary Fig. 4b). Similarly, we obtained concave icosahedra and even multipods with an icosahedral morphology (Supplementary Fig. 4c) when the injection rate for the precursor was increased to 60 ml h^−1^. In this case, surface diffusion was unable to keep up with the deposition of atoms, leaving most of the deposited Pt atoms to accumulate at the vertices.

### Electrochemical measurements

**Figure 2:**
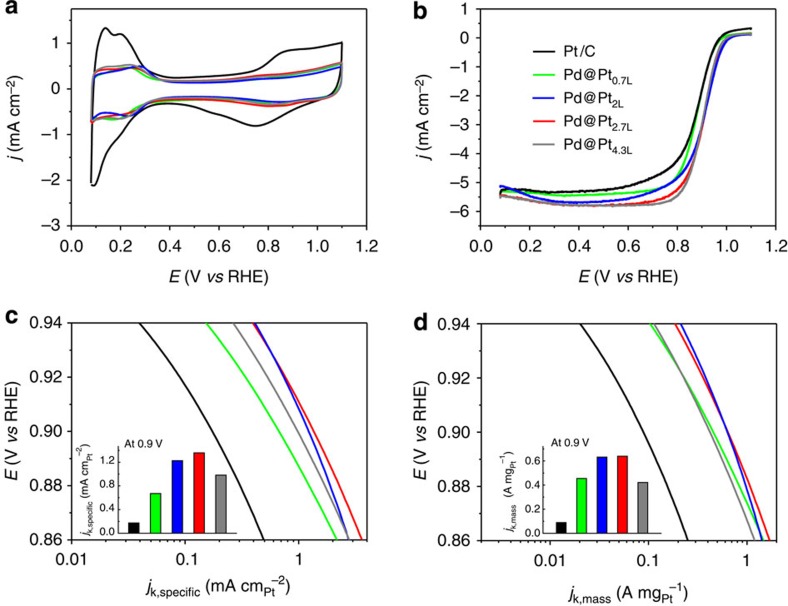
Electrochemical properties of the Pd@Pt_nL_/C catalysts benchmarked against a commercial Pt/C catalyst. (**a**) Cyclic voltammograms recorded from the Pd@Pt_nL_/C catalysts with different number of Pt overlayers and a commercial Pt/C catalyst (denoted as Pd@Pt_0.7L_, Pd@Pt_2L_, Pd@Pt_2.7L_, Pd@Pt_4.3L_ and Pt/C, respectively). (**b**) Comparison of the positive-going ORR polarization curves recorded from the Pd@Pt_nL_/C catalysts with different numbers of Pt overlayers and a commercial Pt/C catalyst. The currents were normalized to the geometric area of the rotating disk electrode. (**c**,**d**) Specific and mass ORR activities given as kinetic current densities (*j*_k_) normalized to the ECSAs and Pt masses of the catalysts, respectively. The colour scheme in (**b**) applies to all other panels.

We then deposited the samples of Pd@Pt_nL_ icosahedra (*n*=0.7, 2.0, 2.7 and 4.3) on carbon to obtain Pd@Pt_nL_/C catalysts and measured their ORR activities using the rotating disk electrode (RDE) method. Figure 2a shows cyclic voltammograms of the Pd@Pt_nL_/C catalysts, together with the data for a commercial Pt/C catalyst. The cyclic voltammogram was recorded at room temperature in a N_2_-saturated 0.1 M HClO_4_ solution at a sweeping rate of 50 mV s^−1^ in the potential range of 0.08–1.1 V versus reversible hydrogen electrode (RHE). From the charges associated with the desorption of hydrogen, we derived the electrochemical active surface area (ECSA) of each catalyst and then normalize the value against the Pt mass to obtain the specific ECSA (Supplementary Fig. 5). Owing to the high dispersion of Pt atoms, the specific ECSA of Pd@Pt_0.7L_/C (73.9 m^2^ g^−1^_Pt_) was much larger than that of the commercial Pt/C catalyst (51.4 m^2^ g^−1^_Pt_). As the number of Pt atomic layers was increased, the specific ECSAs of Pd@Pt_2L_/C, Pd@Pt_2.7L_/C and Pd@Pt_4.3L_/C dropped to 56.3, 47.1 and 42.9 m^2^ g^−1^_Pt_, respectively. However, it is worth noting that these Pd@Pt_nL_/C still had specific ECSAs comparable to that of the Pt/C catalyst even though the Pd@Pt_nL_ particles were almost four times larger than the Pt particles (14.9 versus 3.2 nm). These results support our claim that the dispersion of Pt can be retained when switching to particles with larger sizes, if the Pt atoms are spread into ultrathin shells of only a few atomic layers in thickness.

Figure 2b shows the positive-going ORR polarization curves of the catalysts. The curve was recorded at room temperature in an O_2_-saturated 0.1 M aqueous HClO_4_ solution, with a total metal loading (both Pd and Pt) of 30.6 μg cm^−2^ on the RDE. The kinetic currents of the ORR polarization curves were calculated by following the Koutecky–Levich equation and then normalized against the ECSA and Pt mass to obtain the specific and mass activities (*j*_k,specific_ and *j*_k,mass_), respectively. Relative to the commercial Pt/C, the specific and mass activities of all the Pd@Pt_nL_/C catalysts were greatly enhanced in the potential region of 0.86–0.94 V. According to a recent report by Yang and co-workers^19^, Pt icosahedra with an average diameter of 12 nm had a specific activity of 0.83 mA cm^−2^_Pt_ at 0.9 V. This number is lower than the values we obtained for the Pd@Pt_nL_ (*n*=2, 2.7, and 4.3) icosahedra, albeit very close to the value (0.98 mA cm^−2^_Pt_) for Pd@Pt_4.3L_. Moreover, the *j*_k,specific_ and *j*_k,mass_ of Pd@Pt_nL_/C catalysts showed a volcano-type dependence on the number of Pt atomic layers, with the Pd@Pt_2.7L_/C catalyst exhibiting the highest specific and mass activities (see the insets in Fig. 2c,d). At 0.9 V, the *j*_k,specific_ of the Pd@Pt_2.7L_/C catalyst was 1.36 mA cm^−2^_Pt_, which was 7.8 times higher than that of the Pt/C (0.174 mA cm^−2^_Pt_). As a more critical indicator for the commercialization potential of a Pt-based catalyst, it is worth noting that the *j*_k,mass_ (0.64 A mg^−1^_Pt_) of the Pd@Pt_2.7L_/C was 7.2 times higher than that of the Pt/C catalyst (0.089 A mg^−1^_Pt_).

### DFT calculations

The greatest enhancement in activity was found for the Pd@Pt_2.7L_/C catalyst. The enhancement could be attributed to a combination of both the core-shell structure and the compressive strain imposed on the Pt overlayers due to the presence of extra Pt atoms, which weakens the binding strength of reaction intermediates. This argument is supported by our periodic, self-consistent DFT calculations (generalized gradient approximation PW91 (GGA-PW91)). Previous simulations have shown that there is a tensile strain on the surface of Pt icosahedral nanocrystals relative to the surface of Pt octahedral nanocrystals^23^. Therefore, we first constructed a periodic Pd(111) slab as a model for the surface of the Pd icosahedral seed, in which a tensile surface strain was imposed on the Pd atoms with respect to the bulk-optimized lattice constant. We then placed *n* (*n*=2, 3 and 4) layers of Pt atoms on the stretched Pd(111) surface, denoting the system as Pt_nL_*/Pd(111)_ico_. From the TEM images in Fig. 1e,f, we found that the Pt overlayers contained excess Pt atoms with respect to the case of pseudomorphic deposition. To model this situation, we introduced one extra Pt atom into each Pt overlayer in a (10 × 1) unit cell that represents the Pd(111) surface: that is, each overlayer comprises 11 Pt atoms while each layer of the substrate only contains 10 Pd atoms. The specific Pt/Pd ratio of 11:10 was chosen as a compromise between the experimentally observed ratio (15:14) in Fig. 1e,f, and the computational cost. Although the Pd(111) substrate is under tensile strain, we note that the overall strain on the Pt atoms in the overlayers is compressive relative to the bulk-optimized Pt because of the extra Pt atoms introduced into those layers. This result is significant, as our previous work has shown that the ORR activity of a Pt surface can be greatly enhanced by slightly weakening the binding energy of OH through the introduction of a compressive strain for the Pt lattice or the involvement of ligand effects^13^,^32^,^33^,^34^,^35^. The compressive strain on Pt and the lattice site mismatch caused by the extra Pt atom both lead to surface corrugation in our optimized Pt_nL_*/Pd(111)_ico_ model surfaces (Supplementary Fig. 6), which is consistent with the experimental observation.

**Figure 3:**
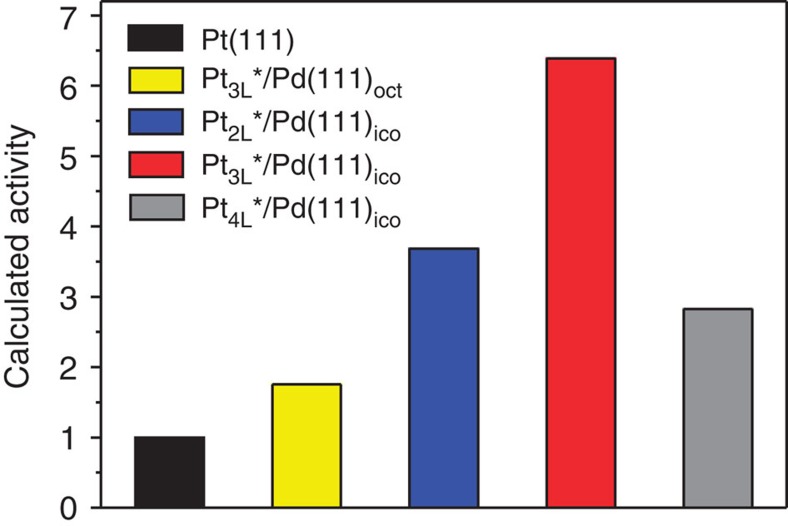
Analysis of the ORR activity of Pt_nL_*/Pd(111)_ico_ slab models using DFT calculations. Relative specific activities of Pd@Pt_nL_ icosahedra at 0.9 V versus (*vs*) RHE calculated using the Pt_nL_*/Pd(111) icosahedral slab models, denoted as Pt_nL_*/Pd(111)_ico_, at a tensile strain of 2.4% for the Pd surface. The result for Pt_3L_*/Pd(111)_oct_ is included for comparison. All values are given relative to the pure Pt(111) surface.

We also calculated the binding energies of OH on the Pt_nL_*/Pd(111)_ico_ model surfaces, in comparison with the results obtained from the model surfaces of Pd@Pt_3L_ octahedra (Table 2). The model for the octahedra is denoted as Pt_3L_*/Pd(111)_oct_, and the respective calculations involved neither tensile surface strain nor excess atoms in the Pt overlayers. Since the precise tensile strain in the (111) facet of a Pd icosahedron was unknown, we calculated the binding energies over a range (1–9%) of strain values. We found that OH bound less strongly to the surface of Pt_3L_*/Pd(111)_ico_ than to the surface of Pt_3L_*/Pd(111)_oct_, as long as the tensile strain in the (111) facet of a Pd icosahedron was 3% or less relative to a Pd octahedron (we used the lattice constant of bulk Pd optimized using PW91 for the Pd octahedron). It is this weakened OH binding caused by compressive strain in the Pt overlayers that leads to a higher specific activity for the Pd@Pt_3L_ icosahedra relative to the octahedral counterpart (Supplementary Table 2). With the application of a simple electrochemical model^36^, we were also able to rationalize the experimentally observed volcano relationship between the ORR specific activity and the number of Pt overlayers. In particular, when the tensile strain in the Pd layers of the Pt_nL_*/Pd(111)_ico_ model was in the range of 2.4–3.0%, we found qualitative agreement between the theoretically predicted values and the experimentally obtained data (Fig. 3). Our calculations indicate that the activity peaked at *n*=3 for the Pt overlayers, in agreement with the most active catalyst (*n*=2.7) identified experimentally. We also note that this range of tensile strain (2.4–3.0%) is very close to the optimal value of 3.2% previously derived for the surface of a Pt icosahedron relative to a Pt octahedron^23^. Corresponding to a range of 2.4–3.0% tensile strain for the underlying Pd lattice, the net compression on the Pt overlayers was calculated to be between 6.1 and 5.0%, respectively. Although this compressive strain is greater than the ‘optimal' value of 2–3% calculated previously for ORR^27^,^28^, we note that much of the strain in the present system is relaxed through the formation of a corrugated surface, which prevents a direct comparison of the magnitude of our compressive strain with the literature values. Overall, our calculation results for Pt_nL_*/Pd(111)_ico_ model slabs demonstrate an important proof of concept that the unique atomic structure on the surface of Pd@Pt_nL_ icosahedra could result in a net compression for the Pt(111) surface, which, in combination with the ligand effect, destabilizes the adsorbed OH and thereby enhances the ORR activity. We do not attempt to reach absolute quantitative agreement with experiments, but demonstrate that our results reproduce experimental trends when the tensile strain on the underlying Pd lattice is within a certain range.

### Thermal stability and catalytic durability

**Figure 4:**
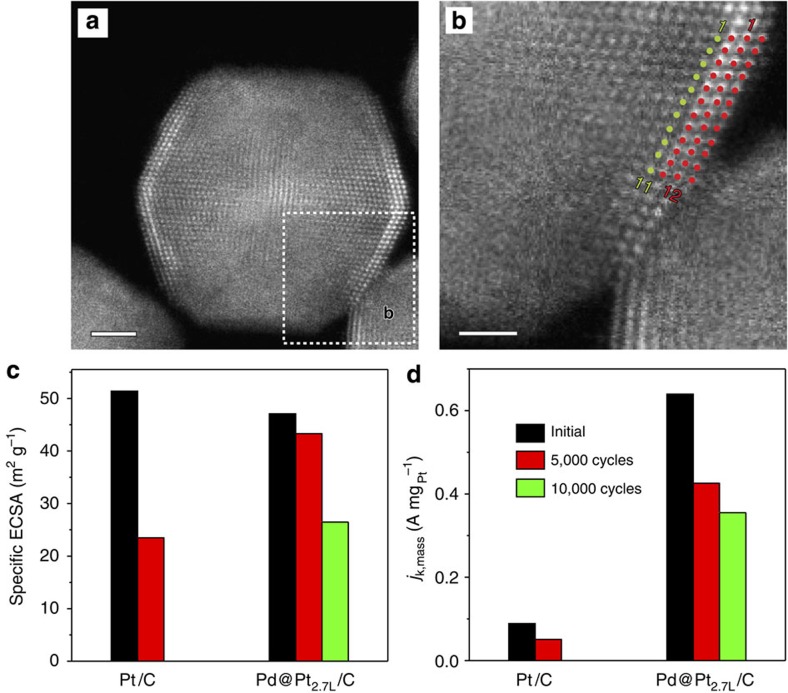
Thermal stability and electrocatalytic durability of the Pd@Pt_2.7L_ icosahedra. (**a**) Atomic-resolution HAADF-STEM image of an individual Pd@Pt_2.7L_ icosahedron along a two-fold symmetry axis after annealing in the electron microscope at 300 °C for 30 min. Scale bar, 2 nm. (**b**) Atomic-resolution HAADF-STEM image taken from the edge marked by a box in (**a**), indicating that the corrugated structure of the Pt shell was retained even after the sample had been heated at 300 °C for 30 min (green dots: Pd atoms; red dots: Pt atoms). Scale bar, 1 nm. A comparison of durability based on (**c**) the specific ECSAs and (**d**) the ORR mass activities at 0.9 V versus RHE for the catalysts before and after accelerated tests.

In addition to the greatly enhanced specific and mass activities, the Pd@Pt_2.7L_ icosahedra exhibited excellent thermal stability and catalytic durability. Under *in situ* heating, the corrugated surface of Pd@Pt_2.7L_ icosahedra was well preserved even after the sample had been heated at 300 °C for 30 min (Fig. 4a,b). The catalytic durability of the Pd@Pt_2.7L_/C sample was evaluated through accelerated tests by applying linear potential sweeps in the range of 0.6–1.1 V at a rate of 0.1 V s^−1^ in an O_2_-saturated 0.1 M aqueous HClO_4_ solution and at room temperature. Judging from both the specific ECSAs and *j*_k,mass_ at 0.9 V, the catalytic durability of the Pd@Pt_2.7L_/C was greatly improved relative to those of the Pt/C (Fig. 4c,d). After 5,000 cycles, the Pd@Pt_2.7L_/C catalyst only exhibited an 8% drop in the specific ECSA, as opposed to a 54% drop for the ECSA of the Pt/C. Even after 10,000 cycles, the specific ECSA of the Pd@Pt_2.7L_/C (26.5 m^2^ g^−1^_Pt_) was still higher than that of the commercial Pt/C (23.5 m^2^ g^−1^_Pt_) only after 5,000 cycles. At 0.9 V, the mass activity of the Pd@Pt_2.7L_/C catalyst after 10,000 cycles still showed fourfold enhancement relative to the pristine Pt/C before durability test.

As suggested in recent publications^37^, the Pt atoms from the Pt counter electrode could be oxidized and dissolved, and then reduced during the potential cycling process. When this occurs, additional Pt nanoparticles can be formed on the carbon support. According to the TEM images shown in Supplementary Fig. 7a,b for the Pd@Pt_2.7L_ icosahedra before and after the durability test, we did not observe the formation of additional Pt nanoparticles on the carbon support. This result indicates that the amount of Pt dissolved from the counter electrode was too little to nucleate and grow into discrete particles. Instead, the small number of Pt atoms could be deposited onto the original catalytic particles. However, the Pd@Pt_2.7L_ icosahedra were found to be transformed into cage-like nanostructures during the durability test due to the selective dissolution of Pd atoms from the cores (Supplementary Fig. 7c,d). This structural transformation makes it very difficult to resolve the possible deposition of extra Pt atoms onto the original Pd@Pt_2.7L_ icosahedra particles.

## Discussion

We have demonstrated a new, cost-effective ORR catalyst based on Pd@Pt_nL_ core-shell icosahedra with well-controlled shell thickness. Owing to the lateral confinement imposed by twin boundaries, the Pt overlayers could only relax along the direction normal to the surface to generate a corrugated structure with compressive strains. When benchmarked against a commercial Pt/C catalyst, the Pd@Pt_nL_ icosahedra exhibited substantial enhancement in both activity and durability towards ORR. These results provide an attractive strategy for designing future catalysts with excellent activity and durability by depositing the active metal as shells of only a few atomic layers on multiply twinned nanocrystals made of another metal, together with an optimized surface structure.

## Methods

### Chemicals and materials

Na_2_PdCl_4_ (99.998%), Na_2_PtCl_6_ (98%), PVP (molecular weight≈55,000), ascorbic acid, KBr and diethylene glycol (DEG, lot no. BCBL4037V) were all obtained from Sigma-Aldrich. Ethylene glycol (lot no. L05B13) was obtained from J. T. Baker. All chemicals were used as received. All aqueous solutions were prepared using deionized water with a resistivity of 18.2 MΩ cm.

### Synthesis of Pd icosahedral seeds

The Pd icosahedral seeds were prepared using a modified protocol^29^. Typically, 80 mg PVP was dissolved in 2.0 ml DEG (hosted in a 20-ml vial) and the solution was heated at 130 °C in an oil bath under magnetic stirring for 10 min. At the same time, 15.5 mg Na_2_PdCl_4_ was dissolved in 1.0 ml DEG and the solution was injected in one shot into the pre-heated solution with a pipette. The vial was capped and continued with heating at 130 °C for 3 h. The product was collected by centrifugation, washed once with acetone and twice with deionized water to remove excess PVP and inorganic ions, and finally re-dispersed in 3 ml ethylene glycol.

### Synthesis of Pd@Pt_nL_ core-shell icosahedra

In a standard procedure, 1.0 ml of the Pd icosahedra (0.59 mg ml^−1^, as determined using ICP-MS), 54 mg KBr, 66 mg PVP, 32 mg ascorbic acid and 9 ml ethylene glycol were mixed in a 50-ml three-neck flask and pre-heated at 110 °C for 1 h. The reaction temperature was then quickly ramped to 200 °C within 10 min. The deposition of Pt atomic layers was initiated by pumping a certain volume of a Na_2_PtCl_6_ solution in ethylene glycol (0.06 mg ml^−1^) into the reaction solution at a rate of 4.0 ml h^−1^. Corresponding to the samples of Pd@Pt_0.7L_, Pd@Pt_2L_ and Pd@Pt_2.7L_ icosahedra, respectively, 4, 12 and 16 ml of the precursor solution was introduced. After the addition of a specific amount of precursor, the reaction solution was kept at 200 °C for another 1 h. The final product was collected by centrifugation, washed once with acetone and twice with ethanol, and re-dispersed in deionized water. We used a similar procedure for the synthesis of Pd@Pt_4.3L_ icosahedra except that 0.5 ml of the Pd icosahedra (0.59 mg ml^−1^) was used and that 21 ml of another precursor solution (0.04 mg ml^−1^ in ethylene glycol) was pumped into the growth solution at a rate of 4.0 ml h^−1^.

### Structural and compositional analyses

TEM images were taken using a Hitachi HT7700 microscope operated at 120 kV by drop casting the nanoparticle dispersions on carbon-coated Cu grids and drying under ambient conditions. High-angle annular dark-field imaging were performed on an aberration-corrected FEI TitanS 80–300 TEM/STEM operated at 300 kV, with a probe convergence angle of 30 mrad and a large inner collection angle of 65 mrad, and a JEOL JEM 2200FS STEM/TEM microscope equipped with a CEOS probe corrector (Heidelberg, Germany) to provide a nominal image resolution of 0.07 nm. The heating experiments were carried out on the FEI microscope using a Protochips Aduro heating stage. To minimize beam irradiation to the sample, a low beam current of 30 pA was used for imaging during heating. The energy-dispersive X-ray spectroscopy analyses were performed in STEM mode using an aberration-corrected JEOL 2200FS electron microscope equipped with a Bruker-AXS silicon drift detector (SDD) detector. The metal contents were measured using ICP-MS (NexION 300Q, PerkinElmer).

### Preparation of the working electrode

First, the Pd@Pt_nL_ icosahedra were loaded on a carbon support (Ketjen Black) with a metal loading content of 20% based on the total mass of Pd and Pt (determined by ICP-MS). The carbon-supported Pd@Pt_nL_ icosahedra were then dispersed in 10 ml of acetic acid and heated at 60 °C for 12 h to clean the surface of the catalytic particles and washed twice with ethanol. After drying, 3.0 mg of the supported Pd@Pt_nL_ catalyst was re-dispersed in a mixture of 1.0 ml of deionized water, 1.0 ml of isopropanol and 40 μl of 5% Nafion under ultrasonication for 20 min. 20 μl of the suspension was deposited on a pre-cleaned RDE (Pine Research Instrumentation) with a geometric area of 0.196 cm^2^ and dried in an oven pre-set to 50 °C. For the Pt/C catalyst (20 wt% 3.2-nm Pt particles on Vulcan XC-72 carbon support, Premetek Co.), the working electrode was prepared using a similar procedure except for the skipping of treatment in acetic acid. The total loadings of metals were 6 μg or 30.6 μg cm^−2^, respectively, for the Pd@Pt_nL_/C (both Pd and Pt) and Pt/C catalysts.

### Electrochemical measurements

Electrochemical measurements were conducted using a RDE connected to a CHI 600E potentiostat (CH Instruments). A leak-free Ag/AgCl/NaCl (3 M) electrode (BASi) was used as the reference. All potentials were converted to values with reference to RHE. The counter electrode was a Pt mesh (1 × 1 cm^2^) attached to a Pt wire. The electrolyte was 0.1 M HClO_4_ prepared by diluting a 70% stock solution (Baker, ACS Reagent grade) with deionized water.

### DFT calculations

All calculations were performed using the VASP code^38^,^39^. The electron–ion interactions were described by projector augmented wave potentials^40^,^41^, and the exchange-correlation functional was described by the generalized gradient approximation (GGA-PW91)^42^. The electron wave function was expanded using plane waves with an energy cutoff of 400 eV.

According to the electron microscopy data in Fig. 1e,f and Supplementary Fig. 2, there existed approximately one extra atom in the Pt overlayer for every 14 atoms in the underlying Pd lattice (a ratio of 15Pt to 14Pd). It should be pointed out that this ratio is based on the cross-sectional image taken along a two-fold symmetry axis and that the result from a two-dimensional analysis, that is, the (111) facet, is not available. With this limitation in mind, we modelled the (111) facets of the Pd icosahedra using stretched (10 × 1) Pd(111) unit cells with seven slab layers. The top five layers were relaxed and successive slabs were separated by five equivalent layers of vacuum. Slabs were constructed with *n* layers of Pt (*n*=2, 3 and 4) placed on top of 7-*n* layers of Pd; we denote these model systems as Pt_nL_*/Pd(111). To include the experimentally observed extra Pt atoms into our model, we added an additional Pt atom into each of the Pt overlayers, that is, (11 × 1) Pt(111) over (10 × 1) Pd(111). This new model is denoted Pt_nL_*/Pd(111)_ico_. We note that the ratio (1.10) of Pt atoms in the overlayers to Pd atoms in the underlying substrate is very close to the ratio (1.07) observed by electron microscopy analysis, and it was chosen to balance computational costs with model realism. The Pt_nL_*/Pd(111)_ico_ model is expected to represent the experimental system reasonably well. As illustrated in Supplementary Fig. 6, the addition of an extra Pt atom to the overlayer result in compression and thus surface corrugation due to the confinement of the Pd substrate.

The surface Brillouin zone was sampled using a 1 × 14 × 1 Monkhorst–Pack *k*-point mesh^43^. This fine *k*-point mesh was chosen because of the very small differences in the binding energy of OH (BE(OH)) observed in this project; binding energies were verified for convergence to 0.005 eV. Geometric optimization was performed until the Hellmann–Feynman forces on atoms were less than 0.01 eV Å^−1^. The dipole correction to the electrostatic potential was included^44^. The optimized lattice constants of Pt and Pd were calculated to be 3.99 and 3.96 Å, respectively, in good agreement with the experimental values of 3.92 and 3.89 Å (ref. 45).

BE(OH) was calculated relative to the clean slabs and gas-phase OH, that is, BE(OH)=*E*_total_–*E*_slab,clean_–*E*_OH,gas_, where *E*_total_ is the total energy of the system with the adsorbate on the slab, *E*_slab,clean_ is the clean slab energy, and *E*_OH,gas_ is the energy of an isolated gas-phase OH species. In addition to the calculation of BE(OH) on Pt_nL_*/Pd(111)_ico_ surfaces, we also performed calculations for slabs of pure Pt(111) using a similar approach (for example, unit cell). The Pd@Pt_3L_ octahedral catalysts were modelled by pseudomorphically depositing three Pt layers over Pd(111), with no extra atoms being added to the Pt overlayers (an illustration is shown in Supplementary Fig. 8). These reference calculations were performed using a (11 × 1) surface unit cell to provide a coverage of Pt that is consistent with the icosahedra model using (11 × 1) Pt in the top layers. Since no extra Pt atoms were included, no surface corrugation was observed in this slab. BE(OH) on all the surfaces examined in this study are shown in Table 2, and the geometries of adsorbed OH on Pt_3L_*/Pd(111)_ico_ and on Pt_3L_*/Pd(111)_oct_ are shown in Supplementary Figs 9 and 10, respectively.

We also calculated the energetics of OH hydrogenation, which has been found to be the rate-determining step in ORR on Pt(111) (refs 46, 47, 48), on these surfaces at 0.9 V (versus RHE) according to the computational standard hydrogen electrode model developed by Nørskov *et al.*, which relates the free energy change (Δ*G*) of a reaction to the cell potential as Δ*G*=Δ*E*+Δ*ZPE*−*T*Δ*S*+|*e*|*U*, where Δ*E* is the change in total energy, Δ*ZPE* is the change in zero-point energies, *T* is the absolute temperature (here 298 K), Δ*S* is the change in entropy, *e* is the electron charge and *U* is the electrode potential. We choose the RHE as the electrochemical reference, at which the reaction *H*_2_←2(*H*^+^+*e*^−^) is at equilibrium at 0 V at standard conditions. Owing to the similar adsorption geometries for all species, in this study, we assume that the entropy and zero-point energy correction is the same on all of the systems.

We calculated the relative activities of these surfaces according to an Arrhenius-type expression, setting the activation energy of OH hydrogenation to the change in free energy of the reaction step^49^. Therefore, since the relative differences in free energy between two surfaces are determined only by differences in binding energies, the rate constant *k*_*i*_ for surface *i* relative to the Pd@Pt_3L_ octahedra catalyst (*k*_3L-oct_) is calculated as 
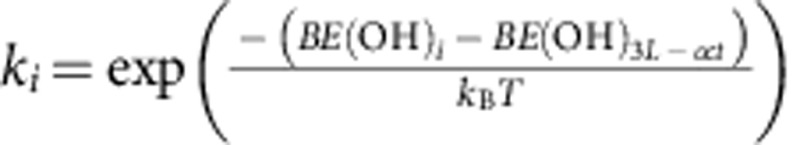
, where BE(OH)_*i*_ is the binding energy of OH on surface *i*, BE(OH)_3L-oct_ is the binding energy on Pt_3L_*/Pd(111)_oct_ and *k*_B_ is the Boltzmann constant. Therefore, any surface that binds OH more weakly than the Pt_3L_*/Pd(111)_oct_ will have a positive value in the numerator of the exponential, and therefore increased activity relative to Pt_3L_ octahedra. The calculated activities for all surfaces in this study are shown in Supplementary Table 2.

## Additional information

**How to cite this article:** Wang, X. *et al.* Palladium–platinum core-shell icosahedra with substantially enhanced activity and durability towards oxygen reduction. *Nat. Commun.* 6:7594 doi: 10.1038/ncomms8594 (2015).

## Supplementary Material

Supplementary Information Supplementary Figures 1-10 and Supplementary Tables 1-2.

## Figures and Tables

**Table 1:** Summary of the Pt shell thicknesses.

**Sample**	***n***	**Wt% of Pt from ICP-MS**	**Wt% of Pt from size and** ***n***_**i**_
Pd@Pt_0.7L_	0.7	11.0	15.5 (*n*_i_=1)
Pd@Pt_2L_	2.0	27.5	27.5 (*n*_i_=2)
Pd@Pt_2.7L_	2.7	34.2	37.1 (*n*_i_=3)
Pd@Pt_4.3L_	4.3	47.0	44.8 (*n*_i_=4)51.1 (*n*_i_=5)

The average number (*n*) of Pt atomic layers was calculated from the ICP-MS data for the Pd and Pt contents in a sample of Pd@Pt_nL_ core-shell icosahedra; the wt% of Pt in the middle column was obtained from the ICP-MS data; and the wt% of Pt in the right column was derived from the size of Pd icosahedra and the integral number (*n*_i_) of Pt atomic layers.

**Table 2:** Binding energies of OH on the surfaces of Pt_nL_*/Pd(111)_ico_ obtained from DFT calculations.

**Slab model**	**Lattice tensile strain for the Pd substrate**			
	**2.0%**	**2.4%**	**2.7%**	**3.0%**
Pt_2L_*/Pd(111)_ico_	−2.638	−2.648	−2.658	−2.666
Pt_3L_*/Pd(111)_ico_	−2.613	−2.634	−2.650	−2.665
Pt_4L_*/Pd(111)_ico_	−2.601	−2.655	−2.680	−2.702

The data were obtained at 1/11 monolayer coverage for the adsorbate. The tensile strain for the icosahedra is given relative to the optimized bulk Pd lattice constant, which was directly used to construct the octahedral models. All binding energies are given in eV. The calculated binding energies on unstrained Pt_3L_*/Pd(111)_ico_ without adding any extra atoms into the Pt overlayers and on pure Pt(111) surface are −2.667 and −2.681 eV, respectively. The binding energies at all strains studied for the Pt_3L_*/Pd(111)_ico_ surfaces were less negative than those for Pt_3L_*/Pd(111)_oct_, accounting for the enhancement in specific activity for the icosahedra relative to the octahedra. We note that the binding energies vary slightly among the eleven unique bridge sites within each reported surface; the standard deviation for these variations is calculated to be 0.018 eV, which is insufficiently large to affect any trends or conclusions reported here.
