# Supplementary material for: Palladium–platinum core-shell icosahedra with substantially enhanced activity and durability towards oxygen reduction
Source: Nat Commun. 2015 Jul 2;6:7594. doi: 10.1038/ncomms8594 (PMC4506534; doi:10.1038/ncomms8594)
Supplement: Supplementary Information — Supplementary Figures 1-10 and Supplementary Tables 1-2. [file ncomms8594-s1.pdf]

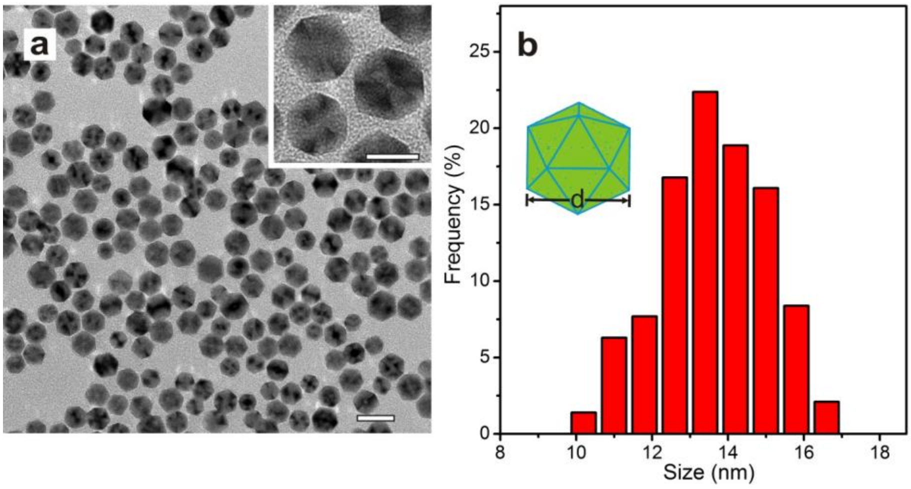

Supplementary Figure 1 | TEM images and the size distribution of Pd icosahedral seeds. (**a**) TEM images of the as-prepared Pd icosahedral seeds. Scale bar, 20 nm. Scale bar in the inset, 10 nm. (**b**) The corresponding histogram of size distribution. As shown by the schematic model in the inset, we defined the diameter of an icosahedron as the distance between two opposite edges.

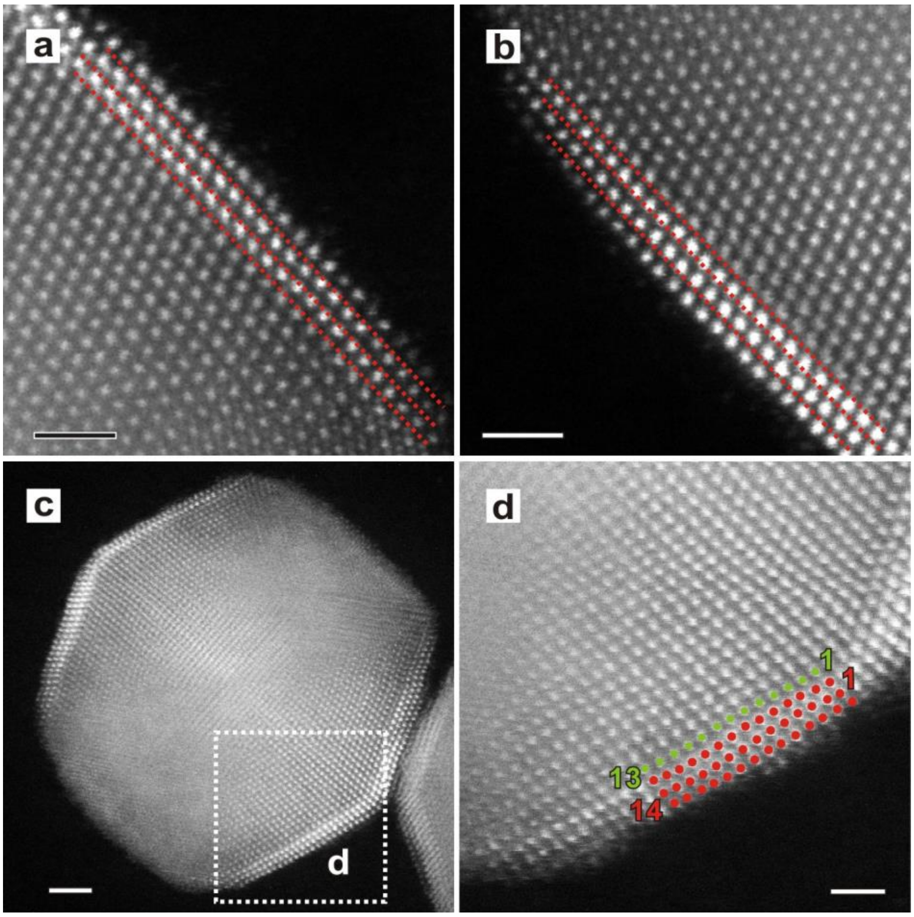

Supplementary Figure 2 | Formation of a corrugated structure by the Pt overlayers. (**a**,**b**) Atomic-resolution HAADF-STEM images taken from the edges marked by boxes in Fig. 1c, showing the formation of a corrugated structure for the Pt overlayers. These two images are duplicates of what are shown in Fig. 1e and 1f, respectively. Scale bars, 1 nm. (**c**) Atomic-resolution HAADF-STEM image of a $Pd@Pt_{2.7L}$ icosahedron (different from the one shown in Fig. 1c) along a 2-fold symmetry axis. Scale bar, 2 nm. (**d**) Atomic-resolution HAADF-STEM image taken from the edge marked by a box in (**c**), revealing a corrugated structure for the Pt overlayers (green dots: Pd atoms; red dots: Pt atoms). Scale bar, 1 nm.

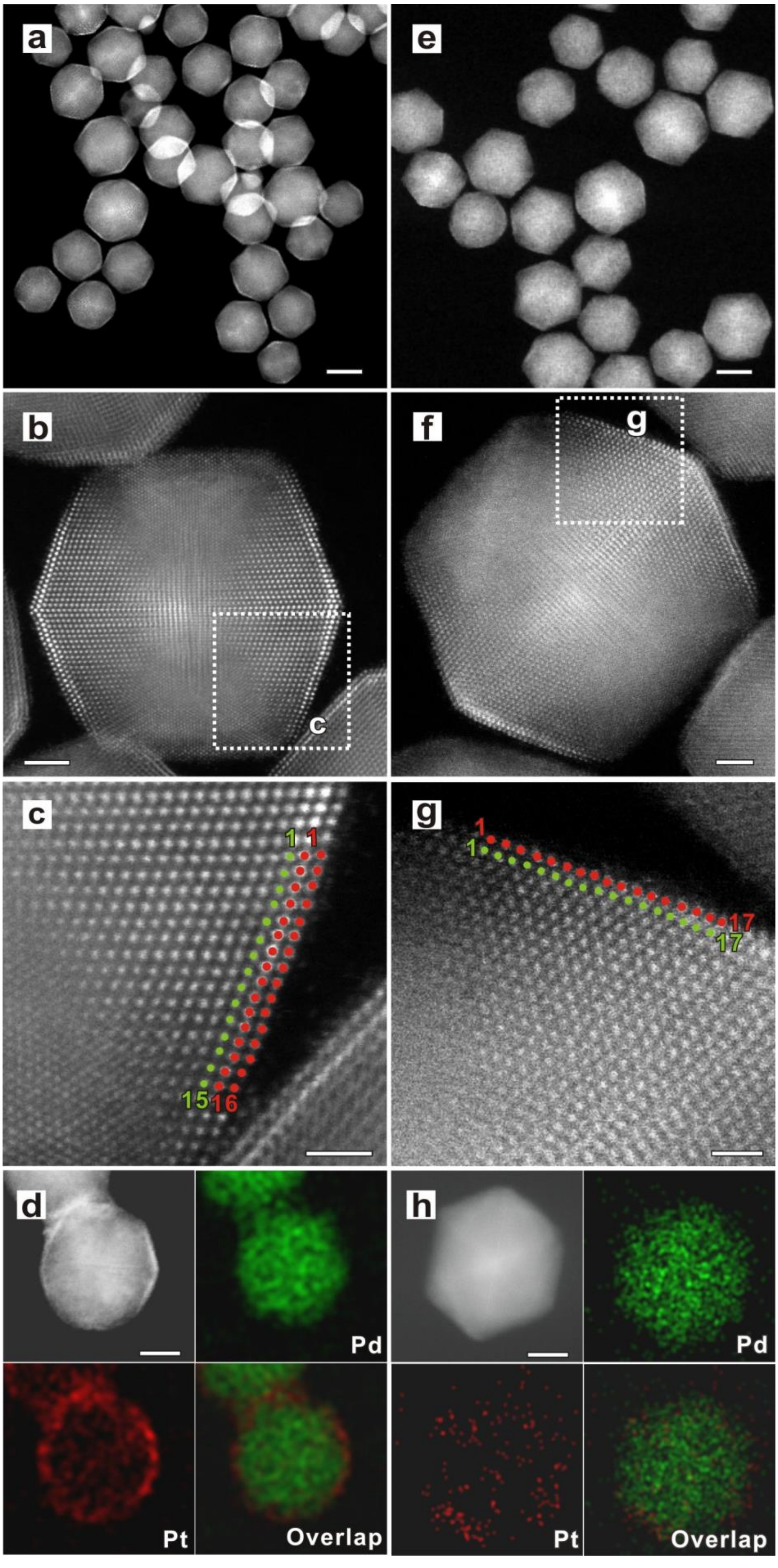

a
e
b
c
f
g
c
1
1
15
16
g
1
1
17
17
d
Pd
Pt
Overlap
h
Pd
Pt
Overlap

Supplementary Figure 3 | Structural and compositional analyses of Pd@Pt_{nL} icosahedra with different numbers of Pt atomic layers. (**a,e**) HAADF-STEM images of Pd@Pt_{2L} and Pd@$Pt_{0.7L}$ icosahedra, respectively. Scale bars, 10 nm. (**b,f**) Atomic-resolution HAADF-STEM images of individual Pd@Pt_{2L} and Pd@$Pt_{0.7L}$ icosahedra, respectively. Scale bars, 2 nm. (**c,g**) Atomic-resolution HAADF-STEM images taken from the edges marked by boxes in (**b**) and (**f**), respectively (green dots: Pd atoms; red dots: Pt atoms). Scale bars, 1 nm. (**d,h**) HAADF-STEM images and EDX elemental mapping taken from individual Pd@Pt_{2L} and Pd@$Pt_{0.7L}$ icosahedra, confirming the Pd@Pt core-shell structure. Scale bars, 5 nm.

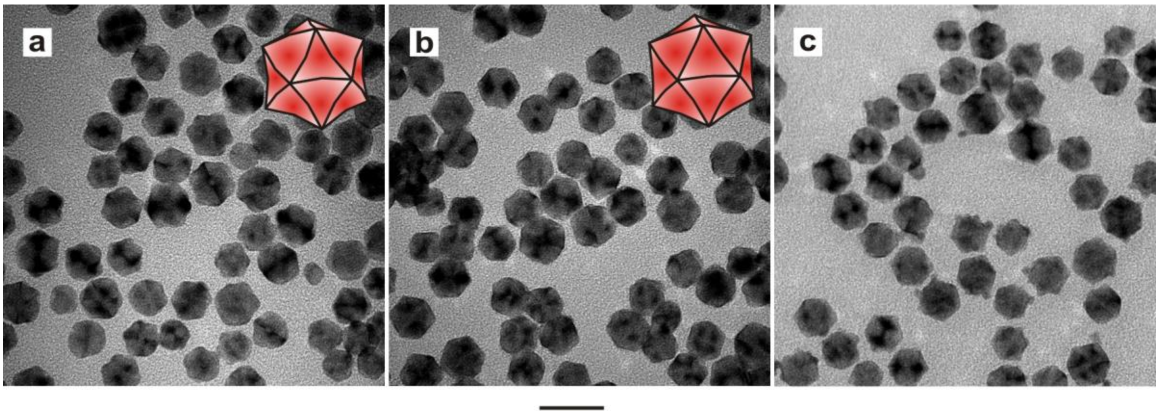

Supplementary Figure 4 | TEM images of Pd@Pt icosahedra obtained under different experimental conditions. (**a**,**b**) TEM images and schematic models of concave Pd@Pt icosahedra synthesized using the standard procedure for the Pd@Pt$_{2.7L}$ icosahedra, except for the decrease of reaction temperature from 200 ºC to (**a**) 120 ºC and (**b**) 140 ºC, respectively. (**c**) TEM image of the Pd@Pt icosahedra synthesized using the standard procedure for the Pd@Pt$_{2.7L}$ icosahedra, except for the use of a faster injection rate (60 mL h^{-1} *vs.* 4 mL h^{-1}) for the precursor. Scale bar, 20 nm.

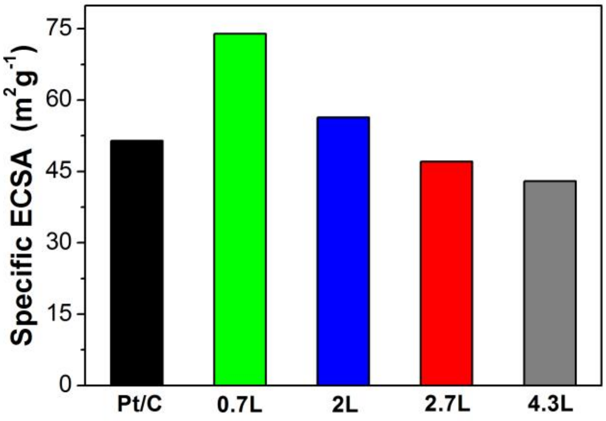

Supplementary Figure 5 | Specific ECSAs of the Pd@Pt$_{nL}$ icosahedra and commercial Pt/C. Specific ECSAs of the Pd@Pt$_{nL}$ icosahedral catalysts with different numbers of Pt atomic layers and the commercial Pt/C (denoted as 0.7L, 2L, 2.7L, 4.3L, and Pt/C) as measured from the charges associated with the desorption of hydrogen in the range of 0.08−0.45 V *vs.* RHE (see the CVs in Fig. 2a).

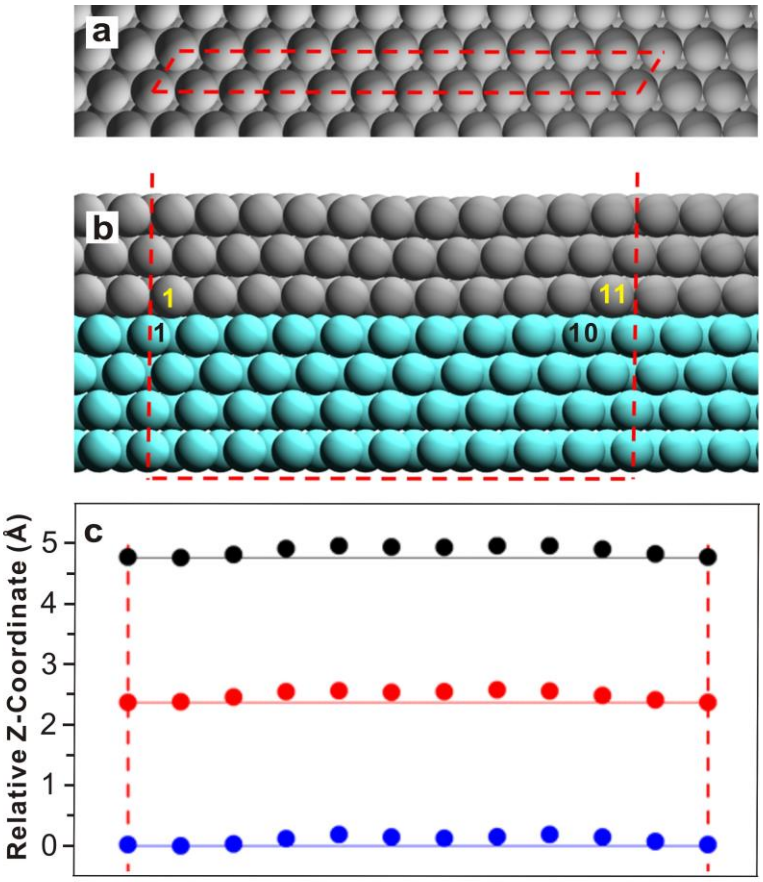

Supplementary Figure 6 | Pt_{3L}*/Pd(111)$_{ico}$ slab model for the Pd@Pt_{3L} core-shell icosahedra. (**a**,**b**) Top view of the (111) surface (**a**) and cross-sectional view (**b**). The unit cell is outlined by the red dashed lines. The icosahedra slab model consists of *n* layers of (11×1) Pt deposited over 7-*n* layers of (10×1) Pd. The Pt atoms are shown in grey while the Pd atoms are in blue. (**c**) Relative z-coordinates of the Pt atoms in the top three layers: black, red, and blue from the top layer to the bottom layer. The z-coordinate varies by as much as 0.2 Å in a given layer, due to compression on Pt layers imposed by the presence of extra Pt atoms in those layers.

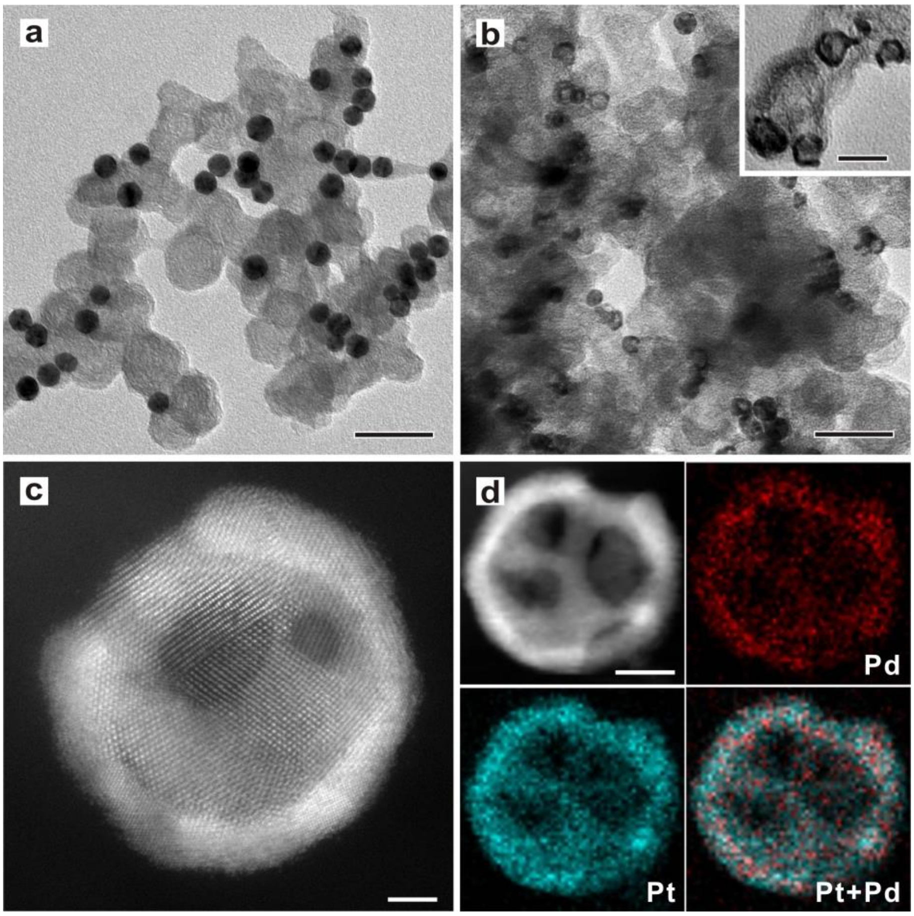

Supplementary Figure 7 | Structural and compositional analyses of the Pd@$Pt_{2.7L}$/C catalyst before and after ORR durability testing. (**a,b**) TEM images of the Pd@$Pt_{2.7L}$/C catalyst (**a**) before ORR test and (**b**) after 10,000 cycles of durability test. Scale bars, 50 nm. Scale bar in the inset, 20 nm. (**c**) Atomic-resolution HAADF-STEM image of a single particle after the durability test, indicating the formation of a hollow structure due to the dissolution of Pd from the core. Scale bar, 2 nm. (**d**) HAADF-STEM image of a single particle after the durability test and the corresponding EDX mapping of Pd and Pt, suggesting the formation of a Pt-Pd alloyed hollow nanostructure during the durability test. Scale bar, 5 nm.

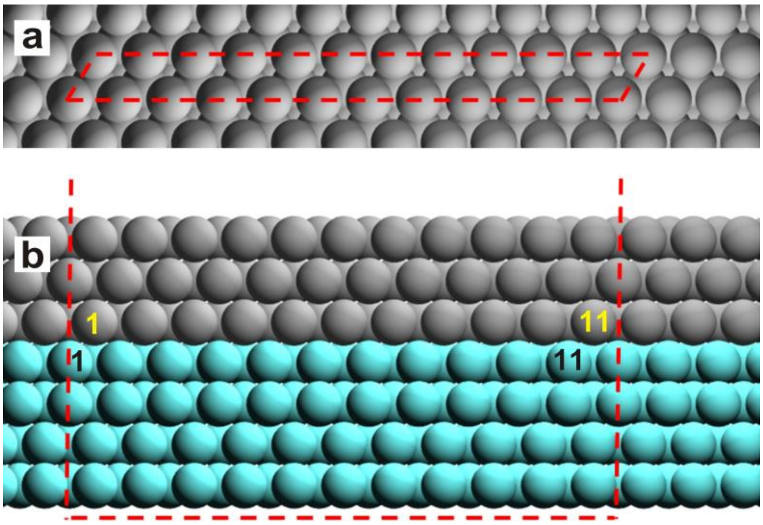

Supplementary Figure 8 | Pt_{3L}*/Pd(111)$_{oct}$ slab model for the Pd@Pt_{3L} octahedra. (**a,b**) Top view of the (111) surface (**a**) and cross-sectional view (**b**). The unit cell is outlined by the red dashed lines. The octahedra slab model consists of *n* layers of (11×1) Pt deposited over 7-*n* layers of (11×1) Pd. The Pt atoms are shown in grey while the Pd atoms are in blue. No surface corrugation is observed in the slab model for octahedra, since no additional Pt atoms were added to the Pt layer as compared to the underlying Pd layer.

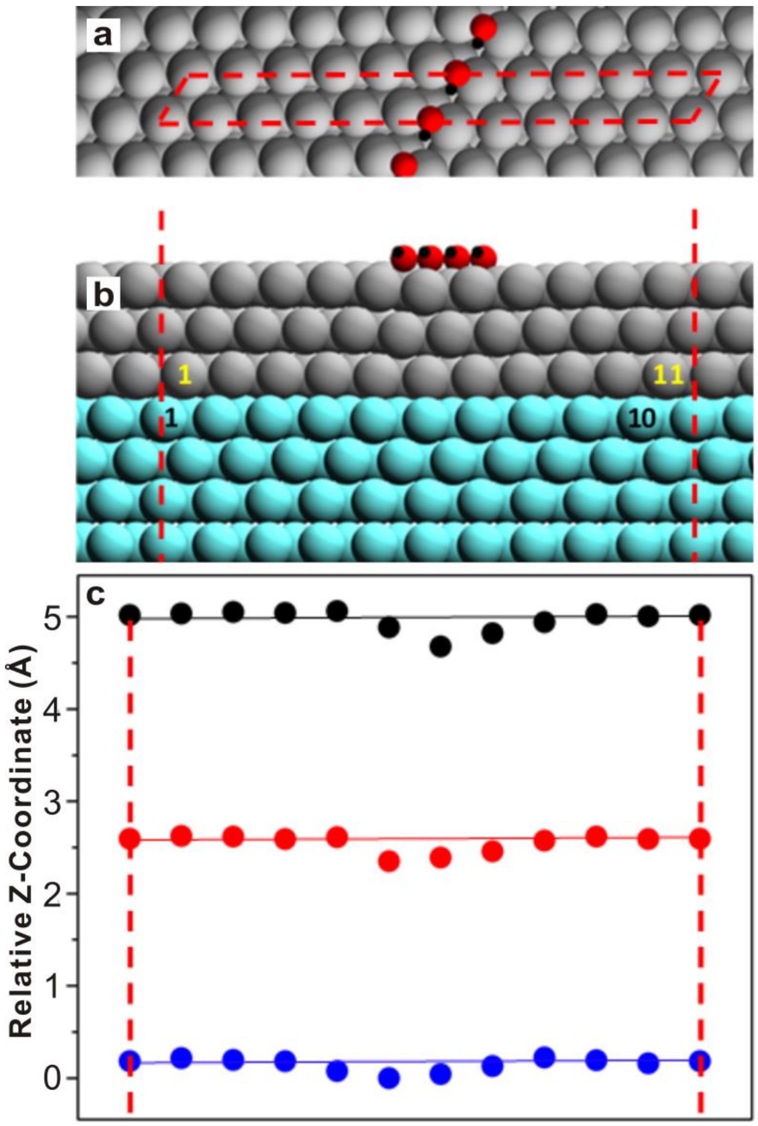

Supplementary Figure 9 | Structure of adsorbed OH on the Pt_{3L}*/Pd(111)$_{ico}$ model. (**a,b**) Top view of the (111) surface (**a**) and cross-sectional view (**b**). The unit cell is outlined by the red dashed lines. Pt atoms are shown in grey, Pd atoms in blue, O atoms in red, and H atoms in black. (**c**) Relative z-coordinates of the Pt atoms in the top three layers: black, red, and blue from the top layer to the bottom layer. In the presence of adsorbed OH, the z-coordinate varies by as much as 0.4 Å in a given layer due to the compression on the Pt lattice.

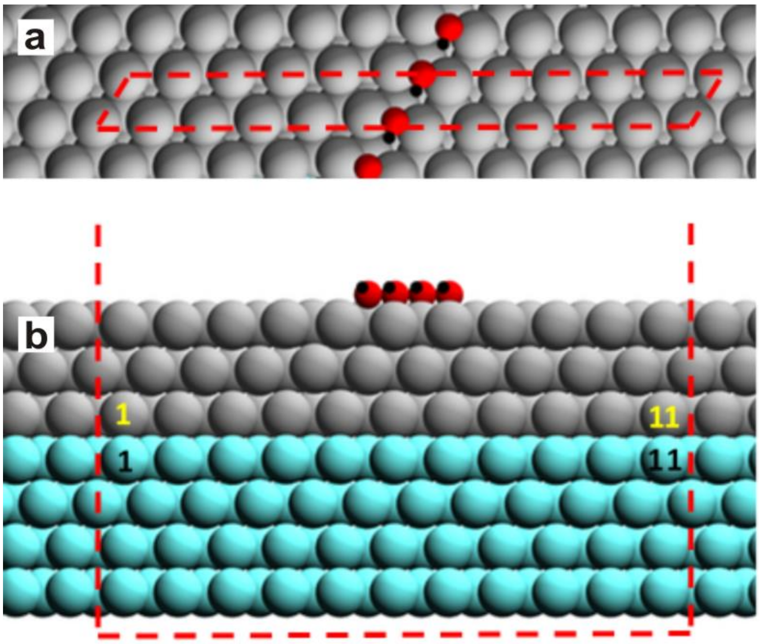

Supplementary Figure 10 | Structure of adsorbed OH on the Pt_{3L}*/Pd(111)$_{oct}$ model. (**a,b**) Top view of the (111) surface (**a**) and cross-sectional view (**b**). The unit cell is outlined by the red dashed lines. Pt atoms are shown in grey, Pd atoms in blue, O atoms in red, and H atoms in black. No substantial surface corrugation is observed in the octahedra model.

Supplementary Table 1. A comparison of the Pt deposition efficiency (%) for the $Pd@Pt_{nL}$ icosahedra as the Pt shells were increased from 0.7 to 4.3 atomic layers.

Sample	Added Pt (mg)	Deposited Pt (mg)	Deposition efficiency (%)
$Pd@Pt_{0.7L}$	0.083	0.073	88.0
$Pd@Pt_{2L}$	0.250	0.225	89.8
$Pd@Pt_{2.7L}$	0.333	0.307	92.2
$Pd@Pt_{4.3L}$	0.292	0.263	90.1

Supplementary Table 2. The relative ORR activities of Pt_{nL}*/Pd(111)$_{ico}$ obtained from DFT calculations.

	Lattice tensile strain of Pd			
**Surface**	**2.0%**	**2.4%**	**2.7%**	**3.0%**
**Pt_{2L}*/Pd(111)$_{ico}$**	5.30	3.68	2.51	1.84
**Pt_{3L}*/Pd(111)$_{ico}$**	14.07	6.39	3.37	1.88
**Pt_{4L}*/Pd(111)$_{ico}$**	22.46	2.82	1.05	0.44

The tensile strain for the icosahedra is given relative to the lattice constant of bulk Pd, which was directly used to construct the octahedral models. All the activities are given relative to pure Pt(111) surface. The unstrained Pt_{3L}*/Pd(111)$_{ico}$, which has no extra Pt atoms in the overlayers is calculated to have a relative activity of 1.75. At all levels of surface strain examined here, the Pt_{3L}*/Pd(111)$_{ico}$ are shown to be more active than the Pt_{3L}*/Pd(111)$_{oct}$.
